# 38.3 ONGOING GERMINAL CENTRE REACTIONS CONTRIBUTE TO N-METHYL-D-ASPARTATE RECEPTOR (NMDAR) ANTIBODY PRODUCTION IN NMDAR-ANTIBODY ENCEPHALITIS

**DOI:** 10.1093/schbul/sby014.156

**Published:** 2018-04-01

**Authors:** Mateusz Makuch, Robert Wilson, Adam Al-Diwani, James Varley, Anne-Kathrin Kienzler, Jennifer Taylor, Patrick Waters, Isabel Leite, Belinda Lennox, Sarosh Irani

**Affiliations:** University of Oxford

## Abstract

**Background:**

Immunoglobulin G (IgG) against the NR1-subunit of the N-methyl-D-aspartate (NMDAR) receptor mediates NMDAR-antibody encephalitis (NMDAR-Ab-E). This multi-stage illness presents with an acute severe psychiatric syndrome, alongside other neurological features, similar to human and animal NMDAR antagonist models. The disease is associated with an ovarian teratoma in around 20% of cases. The cellular immunity underlying this disease is not well understood. While antibody-modifying immunotherapies often promote disease resolution, the illness can be refractory to these treatments correlating with sub-optimal outcomes.

NR1-IgG can be detected several years after clinical resolution, which may be via ongoing germinal centre reactions or the establishment of antibody-secreting cells as long-lived plasma cells in bone marrow niches. These two divergent models implicate use of differing immunotherapies to target these cells. Here we investigate the contribution of ongoing germinal centre reactions to disease progression, potentially informing disease mechanisms and guide targeted immunotherapy.

**Methods:**

We hypothesised that recurrent antigen-driven germinal centre reactions would be associated with active generation of NR1-specific IgM and IgG and NR1-specific circulating B cells. We validated a NR1-IgM cell based assay establishing specificity cut-offs by screening healthy and disease control cohorts alongside a previously collected NMDAR-Ab-E cohort (n=46). Following this we went on to explore the temporal evolution of NR1-IgG and NR1-IgM titres in a prospective cohort (n=12).

To investigate the lymphocyte characteristics, we stimulated ovarian teratoma lymphocytes and peripheral blood mononuclear cells (PBMCs) from multiple time points under varying cytokine conditions to understand whether these circulating cells showed capacity for NR1-IgG and IgM generation.

**Results:**

We found a 43% prevalence rate of NR1-IgM in the historic cohort. We then confirmed that NR1-IgM binding was specific by its selective depletion after anti-IgM precipitation but not with protein G. In the prospective cohort, we noted often high titres of IgM (up to 1:500) most commonly early in the disease but persisting for around 2 years. NR1-IgM levels varied in titre alongside NR1-IgG spikes. Consistently, culture experiments of patient lymphocytes (PBMCs and tumour-derived) produced varying degrees of NR1-IgM and NR1-IgG under conditions associated with B cell proliferation. The NR1-IgG levels correlated with serum NR1-titres suggesting these circulating B cells made a proportional contribution to serum levels.

**Discussion:**

Ongoing germinal centre reactions likely contribute much of the circulating NR1-specific B cell population in NMDAR-Ab-E. Autoimmunisation at these centres represents an as yet unexplored therapeutic target in this and potentially other autoimmune encephalopathies. Regional specificity of these reactions including lymph nodes draining sources of NR1-antigen require further direct evaluation.

